# Smoking cessation after first STEMI enhances infarct healing: a cardiac MRI study

**DOI:** 10.1093/ehjci/jeag087

**Published:** 2026-03-28

**Authors:** Felix Troger, Mathias Pamminger, Martin Reindl, Ivan Lechner, Christina Tiller, Alex Kaser, Philip Lungenschmid, Matthias Schwab, Bernhard Metzler, Sebastian J Reinstadler, Agnes Mayr

**Affiliations:** University Clinic of Radiology, Medical University of Innsbruck, Anichstrasse 35, Innsbruck 6020, Austria; University Clinic of Radiology, Medical University of Innsbruck, Anichstrasse 35, Innsbruck 6020, Austria; University Clinic of Internal Medicine III, Medical University of Innsbruck, Anichstrasse 35, Innsbruck 6020, Austria; University Clinic of Internal Medicine III, Medical University of Innsbruck, Anichstrasse 35, Innsbruck 6020, Austria; University Clinic of Internal Medicine III, Medical University of Innsbruck, Anichstrasse 35, Innsbruck 6020, Austria; University Clinic of Internal Medicine III, Medical University of Innsbruck, Anichstrasse 35, Innsbruck 6020, Austria; University Clinic of Radiology, Medical University of Innsbruck, Anichstrasse 35, Innsbruck 6020, Austria; University Clinic of Radiology, Medical University of Innsbruck, Anichstrasse 35, Innsbruck 6020, Austria; University Clinic of Internal Medicine III, Medical University of Innsbruck, Anichstrasse 35, Innsbruck 6020, Austria; University Clinic of Internal Medicine III, Medical University of Innsbruck, Anichstrasse 35, Innsbruck 6020, Austria; University Clinic of Radiology, Medical University of Innsbruck, Anichstrasse 35, Innsbruck 6020, Austria

**Keywords:** cardiac MRI, infarct healing, myocardial infarction, smoking cessation

## Abstract

**Aims:**

Smoking cessation represents an important step in primary and secondary prevention of myocardial infarction. While there are few data on the extent of initial myocardial damage in current smokers, there are hardly any data on the evolution of the infarct in active smokers and in patients giving up smoking. This prospective study investigated infarct dynamics in patients quitting smoking after ST-elevation myocardial infarction (STEMI).

**Methods and results:**

Overall, 672 revascularized first-time STEMI patients were included. Smoking behaviour was documented at baseline and after 4 months. Cardiac magnetic resonance imaging (CMR) was performed at baseline and after 4 and 12 months, including infarct characteristics evaluation. Major adverse cardiac events (MACE) comprised all-cause death and reinfarction within a median observation period of 3.4 years. Initially, 382 patients (57%) were active smokers, of whom 183 (48%) quit smoking after the infarction. There were no differences in initial infarct size; however, at follow-up CMR examinations, infarct size reduced more in the cessation group compared to continuing smokers (after 12 months: 62% vs. 47%, *P* < 0.001). Additionally, MACE occurred less often in quitters (4%vs. 12%, *P* = 0.005). In multivariable logistic regression, continuing smoking was predictive of reduced infarct healing; in multivariable Cox-regression, it was a marker for MACE, independent of traditional cardiovascular risk markers.

**Conclusion:**

Smoking cessation after the first STEMI appears to be an important factor positively influencing infarct healing. Within the observation period, fewer patients died in the cessation group. In sum, these data provide another valuable argument for quitting smoking, even after an infarction had already occurred.

## Graphical Abstract

**Graphical Abstract:**
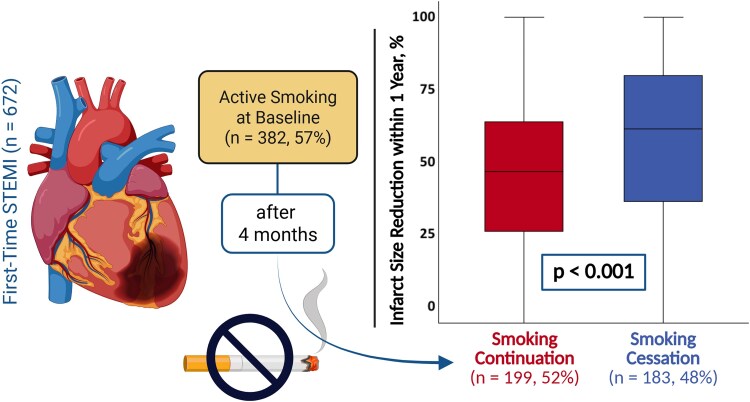
In our cohort, 48% of active smokers quit smoking after myocardial infarction. In these patients, infarct healing within the first year after STEMI was shown to be associated with significantly better infarct healing. *STEMI: ST-elevation myocardial infarction.*

**See the editorial comment for this article ‘CMR evidence of improved infarct healing after STEMI: a call to intensify smoking cessation efforts’, by F. Fortuni and E. Carluccio, https://doi.org/10.1093/ehjci/jeag096.**

## Introduction

Smoking still represents one of the most important cardiovascular risk factors globally, accounting for more than 1.6 million deaths due to coronary heart disease each year (i.e. almost every fifth coronary death).^1^ Thereby, smoking is regarded as the single most important preventable cause of mortality.^2,3^ Smoking cessation, on the other hand, has been shown to bear numerous positive effects concerning cardiac health: amongst others, it reduces arterial stiffness,^4^ increases ‘beneficial’ high-density lipoprotein serum levels^5^ and, lastly, diminishes overall cardiovascular mortality.^6,7^ Even after the occurrence of myocardial infarction, cessation has been shown to decrease the risk of reinfarction,^8^ reduce mortality and improve quality of life.^9^ In terms of imaging, it has been shown that smoking may lead to more severe infarctions concerning the prevalence of intramyocardial haemorrhage (IMH),^10,11^ but apparently has no decisive influence on initial infarct size or the development of microvascular obstructions (MVO).^11–13^ What is more, consistent with the ‘smoker’s paradox’, smoking could even be beneficial regarding negative remodelling after infarction.^11^ Although a single abstract in 2017 hinted at a detrimental effect of smoking concerning infarct healing during the early phase,^14^ data on tissue dynamics of the infarcted area over a longer period are missing. What is more, data on the effect of smoking cessation on cardiac remodelling after STEMI are entirely missing.

Thus, the aims of this current prospective observation study are as follows: a) to compare infarct reduction via cardiac magnetic resonance imaging (CMR) between continuing smokers and patients quitting smoking after first-time ST-elevation myocardial infarction (STEMI) over the first year after infarction; and b) to see whether smoking cessation has an impact on the occurrence of major adverse cardiac events (MACE).

## Methods

### Study population

This prospective study is grounded on the MARINA-STEMI (Magnetic Resonance Imaging In Acute ST-Elevation Myocardial Infarction) study (NCT04113356).

Inclusion criteria included: a) first-time STEMI [defined according to the current guidelines by the European Society of Cardiology,^15^], b) treated by primary percutaneous coronary intervention (PCI) within 24 h following symptom onset. Furthermore, c) all patients with available CMR scans at baseline, after 4 months and after 12 months were analysed for this current investigation.

Exclusion criteria comprised: a) age < 18 years, b) an estimated glomerular filtration rate <30 mL/min/1.73 m^2^, c) a Killip class ≥3 at the time of CMR imaging, d) any history of a previous myocardial infarction or coronary intervention and e) any CMR contraindication (pacemaker, orbital foreign body, cerebral aneurysm clip, manifest claustrophobia, known or suspected contrast agent allergy to gadolinium). A flowchart of included patients allocated to their respective groups is shown in *F**igure 1***.

**Figure 1:**
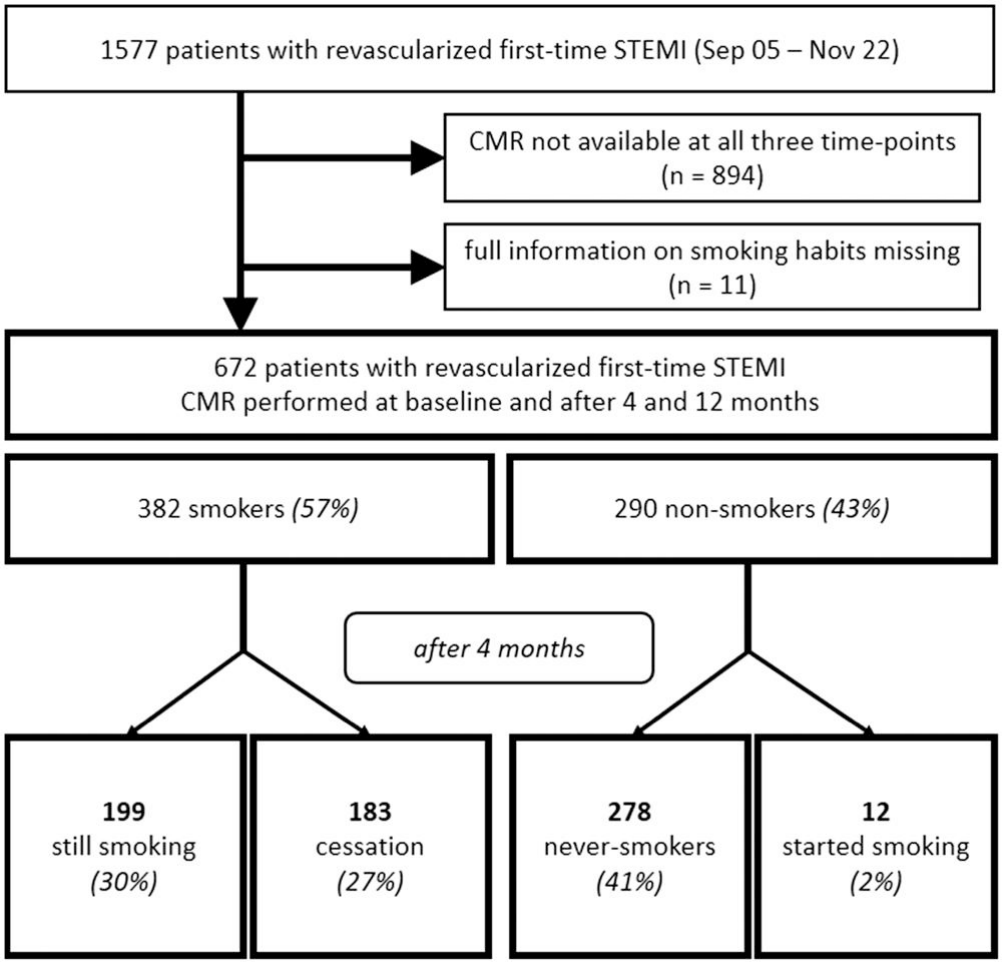
Flowchart depicting study inclusion with smoking status at baseline and after 4 months and the consequential group allocation. *CMR: cardiac magnetic resonance imaging, STEMI: ST-elevation myocardial infarction.*

This study was approved by the local Ethics Committee and conforms to the Declaration of Helsinki. It was conducted following the STROBE guidelines (see supplemental material). No sex- or race/ethnicity-based differences were present.

### Cardiovascular magnetic resonance imaging

All CMR scans were performed on a 1.5 Tesla MAGNETOM AvantoFit scanner (Siemens, Erlangen, Germany) within the first week after PCI and again after 4 and 12 months, each time within a clinical follow-up examination. Regarding image acquisition and post-processing, our standardized research protocol has been applied.^16^

To sum up, functional-volumetric assessment was performed using short-axis cine images acquired by ECG-triggered balanced steady-state free precession bright-blood sequences. Standard software (Circle Cardiovascular Imaging: cvi42, Calgary, Canada) was used for post-processing analyses with semi-automatic detection of left ventricular (LV) endo- and epicardial borders, papillary muscles were excluded from myocardial mass and included in the LV volume. Late gadolinium-enhanced imaging was conducted by administering 0.2 mmol/kg of Gd-DO3A-butriol (Gadovist®, Bayer Vital, Leverkusen, Germany) and obtaining images 15 min thereafter using ECG-triggered phase-sensitive inversion recovery sequences. The infarcted area was defined by hyperenhancement at a threshold of +5 standard deviations above the signal intensity of remote myocardial tissue of the diametrically opposed LV myocardium. Infarct size was expressed as absolute infarct mass (in g, as a product of infarct area, number of obtained slices, interslice gap and specific myocardial mass) as well as percentage of LV myocardial mass yielded in volumetric measurements. MVO was defined as hypoenhancement within the infarct area on late gadolinium-enhanced images. To assess IMH, T2*-mapping was applied using a breath-hold, cardiac-gated gradient echo sequence with 8 echoes obtained in 3 matching short-axis slices before contrast agent administration. Motion correction was applied to reduce corresponding artefacts. IMH was defined as a region of hypointense core within the infarcted area with average T2* values below 20 ms.^17^ Infarct shrinkage after 4 and 12 months is given as a percentage and was calculated according to the formula: 100 * (Infarct Mass**_BASELINE_**—Infarct Mass_FOLLOW-UP_)/Infarct Mass_BASELINE_.

### Clinical follow-up

All currently analysed patients were invited to a clinical follow-up examination according to our study protocol 4 and 12 months after the index event, comprising a standardized questionnaire (including smoking status), physical examination, blood sample analysis and CMR. MACE were thereby defined as a composite of all-cause death and myocardial reinfarction. Newly diagnosed congestive heart failure was defined as symptoms of cardiac decompensation requiring intravenous diuretic treatment or hospitalisation. Before our final statistical analysis, a telephonic follow-up was conducted in patients, in whom the initial event dated back more than 400 days, including the retrieval of long-term MACE data.

### Statistical analysis

SPSS Statistics 31.0 (IBM, Armonk, NY, USA) was used for statistical analyses. All results for continuous variables are expressed as medians with corresponding interquartile range (IQR), categorical variables as absolute numbers and percentages. Differences in categorical variables between two or more groups were tested by the chi-square test. Differences in continuous variables were tested using the Mann–Whitney test (between two groups) and the Kruskal–Wallis test (between more than two groups, with Bonferroni post-hoc testing). A *P*-value <0.05 was considered statistically significant.

Binary logistic regression was performed to evaluate independent markers for enhanced infarct healing (defined as an infarct reduction above the median value). Further, for the analysis of MACE markers, uni- and multivariable Cox Proportional Hazards Survival Regression analysis (Cox-regression) was applied, with the latter being assessed using time-dependent covariate interactions. For both these regression analyses, in addition to smoking status after 4 months, variables with a *P*-value <0.1 in univariable analysis, as well as parameters of clinical significance, were included in our multivariable models. For logistic regression, metric variables were split up into quartiles to improve interpretability. Finally, repeated-measures general linear model analysis with observation interval (baseline vs. 12-month follow-up) as a within-subjects factor and cessation status as a between-subjects factor was conducted in order to further display the effect of cessation on infarct size.

## Results

### Baseline patient characteristics

Overall, 672 patients with first-time STEMI undergoing primary PCI between September 2005 and November 2022 were included in this current analysis, all of whom were acutely referred to the local University Hospital of Cardiology in Innsbruck. Total ischaemia time (i.e. time from pain onset until revascularisation) was 186 min (IQR: 120–320) and did not differ between groups (*P* > 0.050). According to their reported smoking status at baseline and after 4 months, they were allocated to four separate groups: 199 patients (30% of the whole cohort, 52% of smokers) continued smoking (‘still-smokers’); 183 patients (27%, 48% of smokers) quit smoking after the initial event (‘quitters’); 278 patients (41%; 96% of non-smokers) retained non-smoking status (‘never-smokers’); finally, 12 patients (2%, 4% of non-smokers) reported that they started smoking after initial myocardial infarction (‘smoking-starters’). For this study, the main focus was on the comparison of still-smokers and quitters. A respective comparison of these two groups is given in ***Table 1***. T2*-mapping was performed in 479 patients overall (71%). In smokers, median smoking years were 29 years (IQR: 10–46) with approximately one pack per day. In the aftermath of infarction, all patients were treated according to the state of the art. There were no significant medication changes among follow-ups between patients suffering MACE and those who did not (all *P* > 0.05 for aspirin, ACE inhibitors, angiotensin receptor blockers, beta blockers, diuretics and statins). Between quitters and continuing smokers, the sole significant difference in medication changes was a higher proportion of patients taking angiotensin receptor blockers at both follow-ups in the cessation group (11% vs. 2%, *P* = 0.001). At 4-month follow-up, smoking quitters were significantly more often taking angiotensin receptor blockers (15% vs. 6%, *P* = 0.004) and direct oral anticoagulants (9% vs. 2%, *P* = 0.005). There were no differences in medication after 4 months between patients suffering MACE and those who did not. After 4 months, atrial fibrillation occurred more often in smoking quitters (*n* = 4, 2% vs. *n* = 0, 0%, *P* = 0.036).

**Table 1:** Baseline patient characteristics and comparison between still-smokers and quitters

	Total (*n* = 382)	Still Smoking (*n* = 199)	Cessation (*n* = 183)	*P*-value
**Patient Characteristics**				
Age at event, yrs	54 (49–61)	54 (48–61)	55 (50–62)	0.112
Female, *n*(%)	61 (16)	28 (14)	33 (18)	0.291
BMI, kg/m^2^	26 (24–28)	26 (24–28)	26 (24–29)	0.499
Diabetes, *n*(%)	35 (9)	21 (11)	14 (8)	0.326
Hypertension, *n*(%)	151 (40)	87 (44)	64 (35)	0.081
Pack Years	30 (20–45)	35 (20–50)	30 (20–40)	0.**009**
**Infarct Characteristics**				
Culprit Vessel, *n*(%)				
• LAD	147 (38)	69 (35)	78 (43)	0.393
• RCA	169 (44)	95 (48)	74 (40)	
• CX	60 (16)	33 (17)	27 (15)	
• LCA	2 (0.5)	1 (0.5)	1 (0.5)	
• R. intermedius	4 (1)	1 (0.5)	3 (2)	
Ischaemia Time, min	186 (120–320)	193 (120–321)	175 (112–319)	0.546
TIMI pre-PCI ≤2, *n*(%)	355 (93)	187 (94)	168 (92)	0.220
TIMI post-PCI ≤2, *n*(%)	35 (9)	19 (10)	16 (9)	0.761
**Lab at Admission**				
Glucose at admission, mg/dL	127 (111–149)	124 (109–153)	128 (114–144)	0.537
Creatinine, mg/dL	0.9 (0.8–1.1)	0.9 (0.8–1.1)	0.9 (0.8–1.1)	0.111
Peak hs-TnT, ng/mL	4753 (1977–7256)	4720 (2007–7820)	4786 (1823–6895)	0.548
Peak CK, U/L	1861 (959–3365)	1825 (970–3217)	1867 (941–3724)	0.573
Peak NT-proBNP, ng/L	1026 (550–1868)	888 (470–1569)	1175 (636–2033)	0.**006**
Peak CRP, mg/dL	2.2 (1.1–4.3)	2.2 (1.0–4.5)	2.2 (1.1–4.1)	0.755
**Baseline CMR Parameters**				
EF, %	50 (44–57)	50 (44–57)	49 (44–56)	0.417
EDV, ml	167 (141–190)	164 (143–190)	168 (140–190)	0.547
ESV, ml	83 (64–102)	80 (64–101)	85 (64–105)	0.403
Myo Mass, g	125 (109–144)	124 (110–140)	126 (108–147)	0.314
GLS, %	−12 (−14 to −9)	−12 (−14 to −10)	−11 (−14 to −8)	0.213
MVO, *n*(%)	214 (56)	114 (57)	100 (55)	0.603
IMH, *n*(%)	89/272 (33)	35/102 (34)	54/170 (32)	0.664
				0.664
Infarct size, g	18 (8–30)	18 (7–29)	19 (8–31)	0.311
Infarct size, % of LVMM	14 (6–24)	14 (6–23)	15 (7–25)	0.342
Infarct reduction, %, Baseline to 4 months	38 (19–56)	34 (16–53)	40 (24–57)	0.**018**
Infarct reduction, %, Baseline to 12 months	54 (29–73)	47 (26–64)	62 (36–80)	**<**0.**001**

BMI, body mass index; CK, creatine kinase; CRP, C-reactive protein; CX, circumflex artery; EDV, end-diastolic volume; EF, ejection fraction; ESV, end-systolic volume; hs-TnT, high-sensitive troponin T; LCA, left coronary artery; LAD, left anterior descending artery; LVMM, left ventricular myocardial mass; NT-proBNP, n-terminal pro-brain natriuretic peptide; RCA, right coronary artery; TIMI, thrombolysis in myocardial infarction class.

### CMR parameters and infarct shrinkage

At the initial CMR scan, there were no significant differences between still-smokers and quitters concerning functional parameters, the occurrence of MVO and IMH, as well as the absolute and relative infarct size (all *P* > 0.2). Concerning follow-up examinations, the reduction of infarct size showed a significant difference between these two groups in favour of the smoking cessation group (after 4 months: 40% vs. 34%, *P* = 0.018; after 12 months: 62% vs. 47%, *P* < 0.001). Regarding the other functional parameters shown in ***Table 1***, there were no significant differences between groups at 4- and 12-month examinations (all *P* > 0.05). Also, there was no difference in the presence of IMH at follow-up examinations (both *P* > 0.8). LV remodelling (as defined as an increase of end-diastolic volume ≥20% from baseline to 12-month follow-up) did not show any difference between quitters and still-smokers (*P* = 0.977).

### Smoking and infarct healing

In all four groups, infarct size diminished significantly over time between CMR examinations (all *P* < 0.02). There was no significant difference in absolute infarct size between groups at baseline and 4-month scan (*P* = 0.084 and *P* = 0.184, respectively), but infarct size at 12-month follow-up was significantly smaller in the cessation group (*P* = 0.022). A comparison of infarct size reduction in all four groups is shown in *F**igure 2***. A line chart showing the dynamics of mean absolute infarct size in all four groups is shown in *F**igure 3***. An exemplary image displaying different degrees of infarct size evolution in two separate patients (cessation vs. continuation) over 12 months is shown in *F**igure 4***.

**Figure 2:**
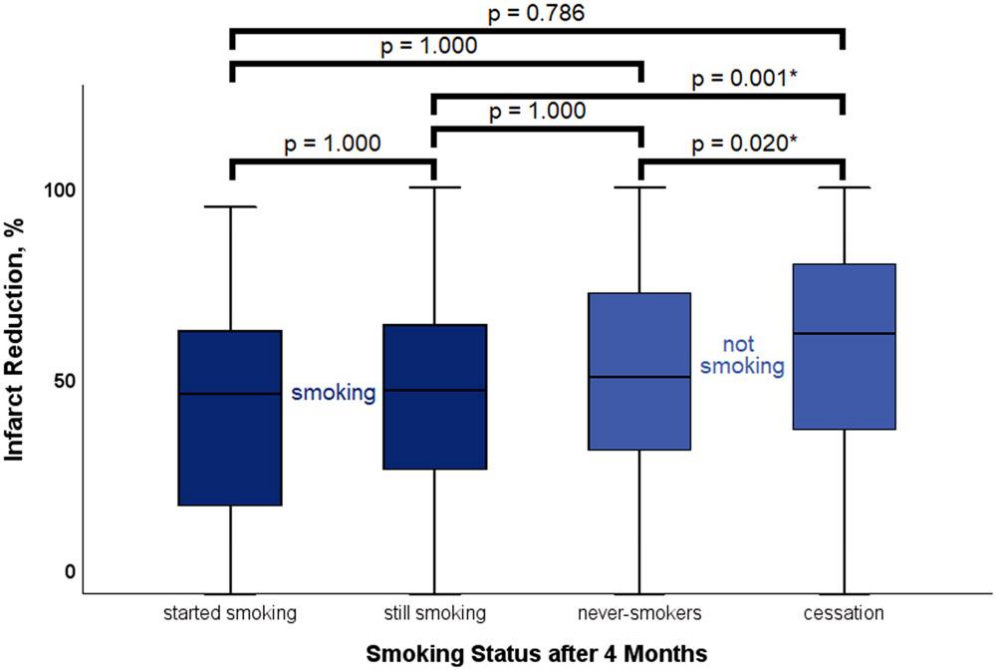
Boxplot diagram illustrating the percentage of infarct shrinkage from baseline to 12-month follow-up by smoking status. * denotes statistical significance.

**Figure 3:**
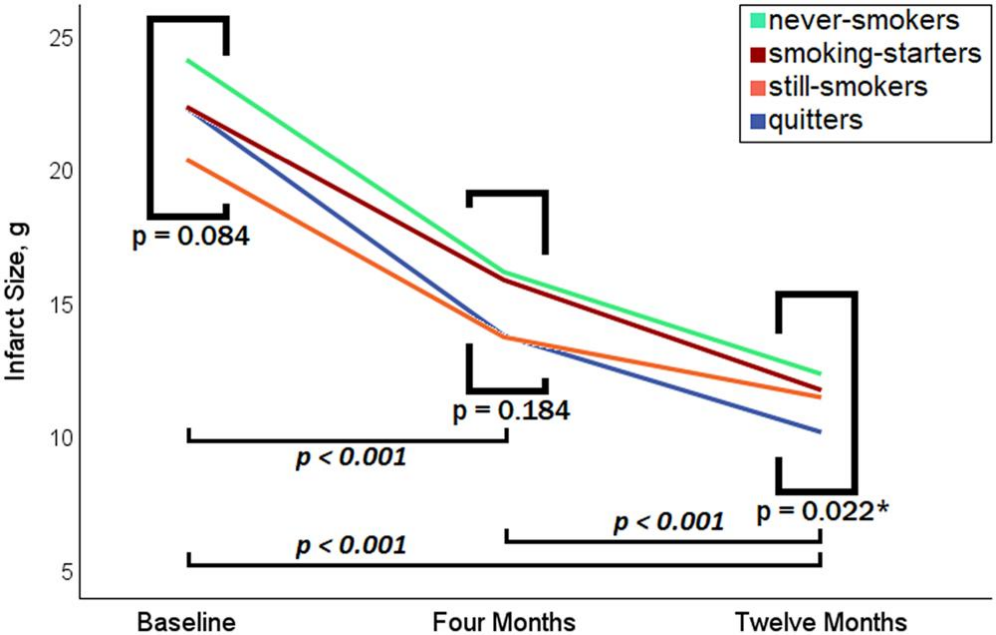
Line chart showing the reduction of mean absolute infarct size over a year according to different smoking status. * denotes statistical significance.

**Figure 4:**
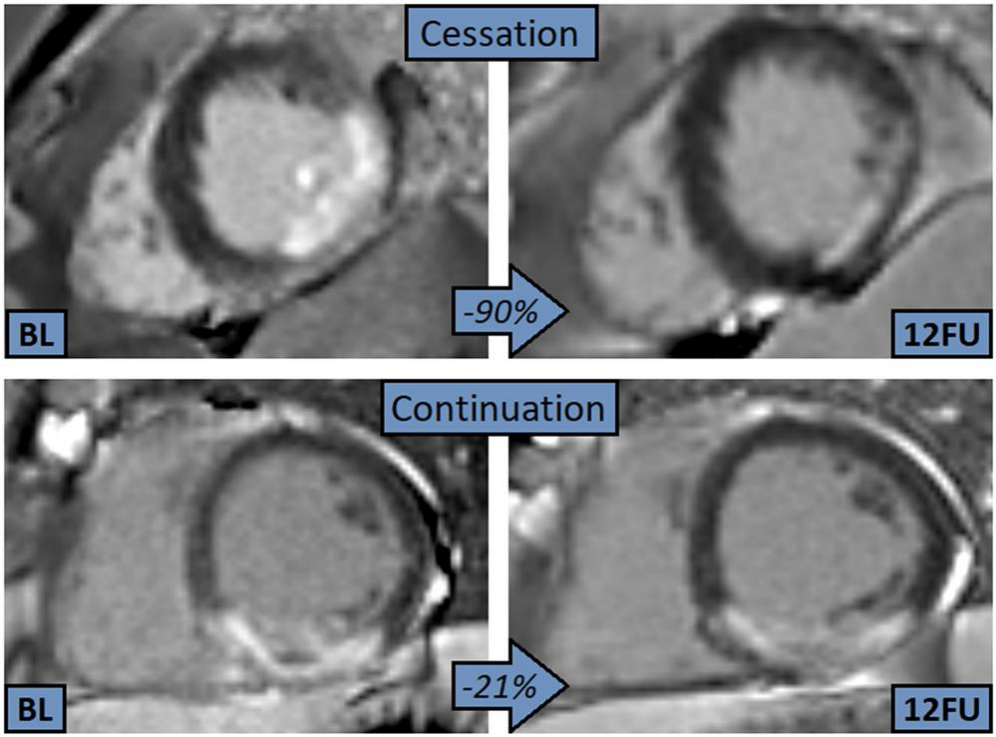
Infarct size evolution over 12 months (left LGE image: baseline CMR, right LGE image: 12 month follow-up) in two patients with markedly different infarct size reduction within the first year after myocardial infarction: the patient in the top row (inferolateral infarction) quitted smoking after the index event, whilst the patient in the lower row (inferoseptal infarction) continued smoking. Percentages represent the relative decrease in infarct size between examinations. *12FU: 12-month follow-up, BL: baseline, CMR: cardiac magnetic resonance imaging.*

Logistic regression analysis determining independent factors for improved infarct healing (defined as more than median infarct reduction, i.e. > 54%, after 12 months) showed smoking cessation to be a significant marker for better infarct healing in univariable analysis [odds ratio (OR): 2.00, 95%CI: 1.31–3.00, *P* = 0.001] as well as in a trivariable model including peak troponin T and presence of MVO (OR: 2.00, 95%CI: 1.31–3.06, *P* = 0.001) as well as in a cumulative model including serum creatinine, peak troponin T, peak creatine kinase, ejection fraction, end-systolic volume and presence of MVO (for cessation: OR: 1.88, 95%CI: 1.21–2.91, *P* = 0.005). In both these multivariable models, besides smoking cessation, troponin T was a significant marker for infarct healing (OR: ≤0.7, *P* < 0.01). Detailed results of this binary logistic regression analysis are shown in ***Table 2***.

**Table 2:** Results of binary logistic regression analysis for the dependent variable ‘improved infarct healing’, defined as infarct reduction above the median value (54% after twelve months) between still-smokers and quitters. Metric variables were stratified by quartiles

(*n* = 382)	Odds Ratio	95% CI	*P*-value
⮚ Univariable			
Smoking cessation	2.00	1.31–3.00	0.**001**
Pack years	1.00	0.99–1.00	0.712
Total ischaemia time, min	0.88	0.73–1.05	0.162
TIMI post-PCI ≤2	0.48	0.26–1.00	0.050
Creatinine, mg/dL	1.15	0.96–1.39	0.138
Peak troponin T, ng/mL	0.69	0.57−0.84	**<**0.**001**
Peak CK, U/L	0.78	0.64-0.94	0.**010**
EF, %	1.19	0.98–1.43	0.073
ESV, ml	0.86	0.72–1.04	0.122
MVO	0.61	0.40-0.92	0.**019**
⮚ **Multivariable**			
***Model I***			
Smoking cessation	2.00	1.31–3.06	0.**001**
Peak troponin T, ng/mL	0.70	0.55-0.89	0.**004**
MVO	0.97	0.57–1.66	0.908
***Cumulative model***			
Smoking cessation	1.88	1.21–2.91	0.**005**
Creatinine, mg/dL	1.20	0.98–1.46	0.077
Peak troponin T, ng/mL	0.62	0.43-0.89	0.**009**
Peak CK, U/L	1.16	0.81–1.66	0.432
EF, %	1.08	0.82–1.43	0.571
ESV, ml	0.99	0.75–1.32	0.963
MVO	1.04	0.59–1.84	0.895

CK, creatine kinase; EF, ejection fraction; ESV, end-systolic volume; MVO, microvascular obstruction.

In a repeated-measures general linear model analysis adjusted for follow-up duration, infarct size decreased significantly from baseline to 12-month follow-up [F(1334) = 298.54, *P* < 0.001, η^2^ = 0.472] with a significant time × cessation interaction [F(1334) = 4.85, *P* = 0.028, η^2^ = 0.014] indicating that the cessation group exhibited greater infarct mass reduction over time.

### Clinical outcomes

Overall, the observation period was 3.4 years (IQR: 2.1–4.6). Within this interval, MACE occurred in 52 patients (8%). When comparing still-smokers to quitters, the first group experienced MACE significantly more often (*n* = 23, 12% vs. *n* = 7, 4%, *P* = 0.015). Further, the prior group experienced all-cause death more often (*n* = 7, 5% vs. *n* = 1, 0.5%). A list comparing MACE data is shown in ***Table 3***. Patients experiencing MACE were significantly more often affected by arterial hypertension prior to infarction (73% vs. 37%, *P* < 0.001) and had more marked infarct size reduction at both follow-ups (*P* < 0.03). A comparison of baseline characteristics between patients suffering MACE and those who did not is shown in the supplement **(see**  Supplementary data online, *Table S1***)**. In the overall cohort, MACE occurred significantly less commonly in the cessation group compared to the other groups pooled (*n* = 7, 4% vs. *n* = 45, 9%, *P* = 0.021).

**Table 3:** Data on MACE, congestive heart failure and LV remodelling according to group allocation

	Total (*n* = 382)	Still Smoking (*n* = 199)	Cessation (*n* = 183)	*P*-value
**MACE, *n*(%)**	30 (8)	23 (12)	7 (4)	0.**005**
**− Death**	10 (3)	9 (5)	1 (0.5)	0.**015**
**− Reinfarction**	24 (6)	17 (9)	7 (4)	0.060
**Congestive Heart Failure, *n*(%)**	19 (5)	7 (4)	12 (7)	0.168
**LV Remodelling after 12 months, *n*(%)**	33	17	16	0.977

LV, left ventricular; MACE, major adverse cardiac events.

In performing Cox-regression for the dependent variable MACE, continuing smoking, hypertension and infarct reduction within the first year below the median were significant predictors in univariable analyses [all with a hazard ratio (HR) of at least 2.5, *P* < 0.04]. In multivariable analyses, continuing smoking was an independent predictive marker for death in models including troponin and ejection fraction (model 1, HR: 2.53, 95%CI: 1.07–6.01, *P* = 0.035) and MVO and initial infarct size (model 3, HR: 2.58, 95%CI: 1.09–6.10, *P* = 0.031), however only by tendency in a model including hypertension and initial infarct size (model 2, HR: 2.08, 95%CI: 0.88–4.93, *P* = 0.097). A list displaying the respective analyses in detail is shown in ***Table 4***.

**Table 4:** Results of Cox regression for the dependent variable MACE in still-smokers and quitters

(*n* = 382)	Hazard Ratio	95% CI	*P*-value
⮚ **Univariable**			
Smoking continuation	2.55	1.08–6.02	0.**033**
Hypertension	5.63	2.78–13.93	**<**0.**001**
Total ischaemia time, min	0.85	0.61–1.18	0.330
TIMI post-PCI ≤2	2.74	1.11–6.77	0.**029**
Peak hs-TnT, ng/mL	1.00	0.72–1.38	0.996
EF, %	0.91	0.65–1.28	0.588
MVO	1.08	0.52–2.27	0.834
IMH	1.75	0.63–4.82	0.281
Infarct size, % of LVMM	1.06	0.76–1.48	0.743
Infarct reduction from baseline to 12 months	0.80	0.57–1.13	0.215
Infarct reduction <54% (median)	2.66	1.12–6.34	0.**027**
⮚ **Multivariable**			
***Model 1***			
Smoking continuation	2.53	1.07–6.01	0.**035**
TIMI post-PCI ≤2	2.58	1.04–6.37	0.**041**
Peak hs-TnT, ng/mL	0.92	0.64–1.32	0.650
EF, %	0.87	0.59–1.26	0.454
***Model 2***			
Smoking continuation	2.08	0.88–4.93	0.097
Hypertension	5.14	2.07–12.78	**<**0.**001**
Infarct size, % of LVMM	1.06	0.75–1.51	0.732
***Model 3***			
Smoking continuation	2.58	1.09–6.10	0.**031**
MVO	0.92	0.35–2.42	0.870
Infarct size, % of LVMM	1.10	0.71–1.70	0.673

Metric variables were stratified by quartiles.

CRP, C-reactive protein; IMH, intramyocardial haemorrhage; LVMM, left ventricular myocardial mass; MACE, major adverse cardiac events.

## Discussion

To the best of our knowledge, this study represents the first to investigate the influence of smoking cessation after STEMI on infarct healing via CMR.

To summarize, our results comprise: a) almost half of the patients with acute STEMI were active smokers and quit smoking after the infarction; b) those patients showed markedly increased infarct reduction and c) suffered MACE less frequently during the observation period compared to those still smoking. In regression analyses, d) cessation was a prognostic marker for improved infarct healing, independent of traditional cardiovascular risk factors, while e) continuing smoking was a marker for MACE.

Although the manifold negative effects of cigarette smoking are widely known by now,^18^ still almost a fifth of people in the European Union were daily smokers as of 2019.^19^ Thereby, smoking remains one of the major causes of premature death worldwide with a total of up to seven million annual deaths.^20^ Contrary to this, smoking cessation has been shown several times to dramatically reduce smoking-related risks and hazards, having an important influence on short- and long-term survival.^21^ In secondary prevention, smoking cessation after myocardial infarction could reduce the risk of reinfarction by 40%^22^ and of cardiovascular death by 45%.^23^ Still, only less than half of these patients seem to be able to accomplish a lasting abstinence after infarction had already occurred,^24^ which, nevertheless, is in line with this current study. Further, an early cessation (i.e. within one year) after infarction has been shown to be even more effective in terms of mortality^25^; as such, all quitters in this current study can be assigned to ‘early cessation’, and the significantly lower mortality in this group is in line with the named study. Fittingly, regarding patients suffering from STEMI, a study on 675 acute STEMI patients concluded that long-term outcomes of those who quit smoking afterwards were comparable to outcomes of non-smokers over an observation period of eight years.^26^ According to a South Korean study, severity of coronary artery disease plays an important role in the sustainability of smoking abstinence,^27^ meaning the number of severely stenotic coronary vessels; in our study, there was no significant difference in the number of affected vessels, although by tendency the quitters initially had a higher percentage of three-vessel disease than the still-smokers (15% vs. 9%, *P* = 0.061). Besides, patients deciding to quit smoking had initially about five pack years less than those who continued in our study. Data on infarct dynamics as assessed via imaging techniques are scarce; nonetheless, an abstract published in 2017 on 109 STEMI patients hinted that smoking was linked to worse healing (as measured via ^99m^Tc tetrofosmin myocardial perfusion imaging) in the very early phase during the first month.^14^ Apart from that, most studies suggested that smoking does not influence initial infarct size.^12,13,28–30^ Still, although studies on the impact of infarct size reduction on prognosis are scarce, a Chinese study by Chen *et al.* in 2025 showed that a better infarct size reduction is associated with a lower prevalence of remodelling.^31^ Besides that, another study by Husser *et al*. showed that patients with a larger infarct size measured 6 months after the infarction still had an increased risk of MACE.^32^ Interestingly, in our study, infarct reduction was highest in the cessation group (with an extreme case illustrated in *F**igure 4***), even surpassing the never-smokers. Although reasons for this cannot be given with certainty, a sensible explanation may lie in the fact that smoking for years might lead to a higher presence of coronary collaterals (due to some kind of ‘preconditioning’) that eventually could be responsible for a quicker and more marked recovery of the infarcted area,^33^ once the continuing noxa of nicotine drops out. Furthermore, the continuously elevated stress level due to oxidative stress suddenly normalizes after cessation (amongst others, resulting in an increase in glutathione),^34,35^ which might in return additionally boost healing. A similarly positive effect could have been shown in general wound healing in a study deducing that the benefits of cessation primarily start at the level of tissue microenvironment (i.e. tissue oxygenation and metabolism) and subsequently continue mid- and long-term at the level of inflammatory and reparative cell functions.^36^ Conversely, as can be seen in *F**igure 3***, at all three CMR examinations, the never-smokers always had the largest infarct size (although not significantly at baseline and 4 months). The harms of starting smoking (anew) after infarction can, due to the small group size, only be estimated, but even then, it is evident that this group had the worst infarct healing, which indicates that starting smoking after myocardial infarction had already occurred appears to be one of the worst options. Of note, arterial hypertension has been shown to be a considerable predictive marker of MACE, e.g. surpassing infarct size in logistic regression analysis. While the negative impact of antecedent hypertension in STEMI has been prominently shown,^37,38^ there are controversial data on the last and final effect of infarct size and MVO on outcome after STEMI. While some authors list them to be predictive in terms of MACE,^39^ other studies could not reproduce this association.^40,41^ Finally, in our study, there was no difference between continuing and quitting smoking concerning adverse LV remodelling, on which, however, smoking has been reported to be of beneficial effect as part of the ‘smoker’s paradox’.^11^ While the latter’s pathophysiological background remains unclear, it is currently mainly believed to be a compound of favourable patient characteristics, different clot properties and, again, a kind of ‘pre-conditioning’ triggered by smoking.^13,42–44^ Nevertheless, the proclaimed smoker’s paradox generally does not apply to long-term outcomes after myocardial infarction.^45^ Eventually, whilst smoking cessation is an effective cornerstone in secondary prevention after STEMI, decisive steps to enhance long-lasting cessation include physician advice, early initiation of evidence-based pharmacotherapy combined with behavioural support and structured follow-up, acknowledging tobacco dependence as a chronic, relapsing condition requiring sustained intervention.^46,47^ Beyond conventional counselling and pharmacological support, smoking cessation after STEMI may be further enhanced by digital health strategies that improve patient engagement and adherence, including telemedicine, mobile health applications, remote monitoring, and digital education platforms, which can support sustained behavioural change and compliance with secondary prevention therapies.^48,49^ In summary, quitting smoking was amongst the strongest predictors of a positive infarct healing in our study, and what is more, it was strongly linked to a beneficial clinical outcome. While the road to a sustainable cessation is undoubtedly rocky, this study provides another important argument to follow it through nonetheless.

### Limitations

We declare that this current study has some limitations: first, regarding smoking behaviour prior to the infarction, it was not sharply distinguished between non- and never-smokers due to these data not having been fully collected in detail; however, an additional subdivision could have potentially added some value to this current analysis. Further, due to the inclusion criteria of this current study (i.e. CMR after 12 months), patients suffering death within the first year were automatically excluded, which is why early mortality cannot be assessed in this study. Then, an even longer observation period could have strengthened this study’s conclusions due to an expected increase in MACE numbers over time. Furthermore, T2*-mapping was not performed in all patients due to its non-availability at our research site before November 2015. Nevertheless, a complete analysis of the presence of intramyocardial iron would have been desirable to further characterize myocardial damage. Lastly, regarding modern trends, this study did not differentiate between ‘conventional’ cigarette smoking and newer forms of smoking such as e-cigarettes or vaporizers. Although the percentage of patients in our cohort using this latter group can be assumed to be quite low, we cannot give definite numbers. Yet, due to the growing use of these products, the health effects of these devices would be of further interest, and respective outcome data are desperately needed.

### Conclusion

In patients having suffered STEMI, smoking cessation appears to be a sensible intervention linked to markedly improved infarct healing within the first year after the index event, and a lower occurrence of MACE during the observation period. This adds another potent argument for quitting smoking, even after an infarction has already taken place. Further, a missing association of continuing smoking with the occurrence of LV remodelling strengthens the critique of the ‘smoker’s paradox’.

## Supplementary Material

jeag087_Supplementary_Data

## Data Availability

The datasets used and/or analysed during the current study are available from the corresponding author on reasonable request.
