# Supplementary material for: Smoking cessation after first STEMI enhances infarct healing: a cardiac MRI study
Source: Eur Heart J Cardiovasc Imaging. 2026 Mar 28;27(6):1287–96. doi: 10.1093/ehjci/jeag087 (PMC13222720; doi:10.1093/ehjci/jeag087)
Supplement: jeag087_Supplementary_Data [file jeag087_supplementary_data.docx]

Supplemental Data

|  | **No MACE (n = 349)** | **MACE (n = 30)** | **p-value** |
| --- | --- | --- | --- |
| **Patient Characteristics** |  |  |  |
| Age at event, yrs | 54 (49-61) | 54 (48-64) | .810 |
| Female, n(%) | 57 (16) | 3 (10) | .362 |
| BMI, kg/m² | 26 (24-29) | 26 (24-28) | .274 |
| Diabetes, n(%) | 32 (9) | 2 (7) | .645 |
| Hypertension, n(%) | 128 (37) | 22 (73) | **<.001** |
| **Infarct Characteristics** |  |  |  |
| Culprit Vessel, n(%) |  |  |  |
| - LAD | 136 (39) | 9 (30) | .463 |
| - RCA | 150 (43) | 18 (60) |  |
| - CX | 57 (16) | 3 (10) |  |
| - LCA | 2 (.5) | 0 (0) |  |
| - R. intermedius | 4 (1) | 0 (0) |  |
| Ischemia Time, min | 192 (120-331) | 146 (95-246) | .090 |
| TIMI pre-PCI ≤2, n(%) | 325 (93) | 27 (90) | .440 |
| TIMI post-PCI ≤2, n(%) | 29 (83) | 6 (20) | **.035** |
| **Lab at Admission** |  |  |  |
| Glucose at admission, mg/dl | 127 (112-149) | 125 (110-136) | .352 |
| Creatinine, mg/dl | 1.0 (.9-1.1) | 1.1 (.9-1.2) | .433 |
| Peak hs-TnT, ng/ml | 4709 (2016-7213) | 4824 (1067-8049) | .994 |
| Peak CK, U/l | 1863 (974-3342) | 1671 (597-3909) | .872 |
| Peak NT-proBNP, ng/l | 1026 (557-1900) | 1042 (337-1606) | .411 |
| Peak CRP, mg/dl | 2.2 (1.1-4.3) | 2.3 (1.1-5.2) | .952 |
| **Baseline CMR Parameters** |  |  |  |
| EF, % | 50 (44-56) | 51 (42-58) | .863 |
| EDV, ml | 167 (140-190) | 158 (144-191) | .848 |
| ESV, ml | 83 (64-102) | 77 (67-109) | .947 |
| Myo Mass, g | 125 (109-144) | 137 (107-146) | .538 |
| GLS, % | -12 (-14 to -9) | -11 (-14 to -8) | .568 |
| MVO, n(%) | 195 (56) | 17 (57) | .933 |
| IMH, n(%) | 81/255 (32) | 7/15 (47) | .251 |
|  |  |  |  |
| Infarct Size, g | 18 (8-30) | 21 (8-29) | .698 |
| Infarct Size, % of LVMM | 14 (6-24) | 15 (6-23) | .657 |
| Infarct Reduction, %, Baseline to 4 Months | 38 (19-57) | 25 (-19 to 43) | **.026** |
| Infarct Reduction, %, Baseline to 12 Months | 55 (31-74) | 39 (23-58) | **.023** |

**Supplemental Table S1.** Comparison of baseline characteristics compared between patients experiencing all-cause death and those who did not during observation period in patients with an active smoking status at the time of the index event. *BMI: body mass index, CK: creatine kinase, CRP: C-reactive protein, CX: circumflex artery, EDV: end-diastolic volume, EF: ejection fraction, ESV: end-systolic volume, hs-TnT: high-sensitive troponin T, LCA: left coronary artery, LAD: left anterior descending artery, LVMM: left ventricular myocardial mass, NT-proBNP: N-terminal pro-brain natriuretic peptide, RCA: right coronary artery, TIMI: thrombolysis in myocardial infarction.*
